# Situating socio-hydrogeology: Towards a transdisciplinary understanding of groundwater monitoring practices in western India

**DOI:** 10.1007/s10040-026-03071-w

**Published:** 2026-04-21

**Authors:** Dhaval Joshi

**Affiliations:** School of Society and Environment, Queen Mary University of London, London, UK

**Keywords:** Groundwater monitoring, Socio-hydrogeology, Groundwater management, Situated knowledge, India

## Abstract

With an increasing emphasis on participatory and citizen-led knowledge initiatives for addressing challenges in improving groundwater governance, socio-hydrogeology is well poised to reveal the complex groundwater–society relationships. Socio-hydrogeological initiatives, while gaining traction among groundwater scholars and practitioners, are embedded in positivist, quantitative approaches, led by hydrogeologists, rarely integrating insights from critical social sciences. Drawing on recent work in critical water studies and socio-hydrology, this paper calls for ‘situating’ socio-hydrogeology by advancing a transdisciplinary understanding of groundwater monitoring practices in the western Indian state of Maharashtra. The paper is based upon a qualitative inquiry into practices of groundwater knowledge production with groundwater officials and farming communities. The paper’s findings demonstrate the interplay and integration between scientific practices of groundwater monitoring and alternative, place-based ways of knowing groundwater. It underlines the value of ‘being in the field’ over technological, distant forms of monitoring that are being mobilised by international, national and regional agencies. It reveals how groundwater officials navigate challenges in everyday practices of monitoring to move ‘beyond hydrogeology’—involving decisions made ‘off the record’ that are shaped by concerns of avoiding bureaucratic burdens. Lastly, groundwater understanding in farming communities is embedded in their cultural and material relationships with groundwater and agriculture, thus offering alternative forms of knowing groundwater. Collectively, these findings advance a case for situating socio-hydrogeology towards a grounded, place-based transdisciplinary enterprise that informs just transformations for groundwater sustainability.

## Résumé

Face à l'importance croissante accordée aux initiatives participatives et citoyennes de partage des connaissances pour relever les défis liés à l'amélioration de la gouvernance des eaux souterraines, la socio-hydrogéologie est idéalement placée pour révéler la complexité des relations entre les eaux souterraines et la société. Bien que les initiatives socio-hydrogéologiques gagnent en popularité auprès des chercheurs et des praticiens du domaine, elles s'appuient sur des approches positivistes et quantitatives, menées par des hydrogéologues, et intègrent rarement les apports des sciences sociales déterminantes. S'appuyant sur des travaux récents en études critiques sur l'eau et en socio-hydrologie, cet article propose de « positionner » la socio-hydrogéologie en développant une compréhension transdisciplinaire des pratiques de surveillance des eaux souterraines dans l'État du Maharashtra, dans l'ouest de l'Inde. L'article repose sur une enquête qualitative menée auprès des responsables de la gestion des eaux souterraines et des communautés agricoles, portant sur les pratiques de production de connaissances relatives aux eaux souterraines. Les résultats de cette étude démontrent l'interaction et l'intégration entre les pratiques scientifiques de surveillance des eaux souterraines et les modes de connaissance alternatifs des eaux souterraines, ancrés dans le territoire. Ils soulignent la valeur du travail de terrain par rapport aux formes de surveillance technologiques et distantes mobilisées par les agences internationales, nationales et régionales. Ils révèlent comment les responsables des eaux souterraines surmontent les difficultés rencontrées dans leurs pratiques quotidiennes de surveillance pour aller au-delà de l'hydrogéologie, notamment par le biais de décisions prises de manière informelle, motivées par la volonté d'éviter les lourdeurs bureaucratiques. Enfin, la compréhension des eaux souterraines au sein des communautés agricoles est intrinsèquement liée à leurs relations culturelles et matérielles avec ces ressources et l'agriculture, offrant ainsi d'autres formes de connaissance. Collectivement, ces résultats plaident pour une socio-hydrogéologie s'inscrivant dans une démarche transdisciplinaire ancrée dans le territoire, contribuant à des transformations justes pour une gestion durable des eaux souterraines.

## Resumen

Con un énfasis creciente en iniciativas de conocimiento participativas y lideradas por ciudadanos para abordar los desafíos en la mejora de la gobernanza de las aguas subterráneas, la sociohidrogeología está bien posicionada para revelar las complejas relaciones entre el agua subterránea y la sociedad. Las iniciativas socio-hidrogeológicas, aunque ganan terreno entre los estudiosos y profesionales de las aguas subterráneas, están integradas en enfoques positivistas y cuantitativos, liderados por hidrogeólogos, que rara vez integran conocimientos de ciencias sociales críticas. Basándose en trabajos recientes en estudios críticos del agua y sociohidrología, este artículo aboga por 'situar' la sociohidrogeología avanzando en una comprensión transdisciplinar de las prácticas de monitoreo de aguas subterráneas en el estado occidental indio de Maharashtra. El artículo se basa en una investigación cualitativa sobre las prácticas de producción de conocimiento sobre el agua subterránea con funcionarios de aguas subterráneas y comunidades agrícolas. Los hallazgos del artículo demuestran la interacción e integración entre las prácticas científicas de monitoreo de las aguas subterráneas y las formas alternativas basadas en el lugar de conocer el agua subterránea. Subraya el valor de 'estar en el terreno' frente a las formas tecnológicas y distantes de monitoreo que están siendo movilizadas por agencias internacionales, nacionales y regionales. Revela cómo los responsables de aguas subterráneas navegan los desafíos en las prácticas cotidianas de monitoreo para ir 'más allá de la hidrogeología', es decir, decisiones tomadas 'off the record' que están influenciadas por la preocupación de evitar cargas burocráticas. Por último, la comprensión del agua subterránea en las comunidades agrícolas está arraigada en sus relaciones culturales y materiales con la agricultura, ofreciendo así formas alternativas para su conocimiento. En conjunto, estos hallazgos avanzan un argumento para situar la sociohidrogeología hacia una organización transdisciplinar con base sólida y en el lugar que impulse transformaciones justas para la sostenibilidad de las aguas subterráneas.

## Abstract (zh)

随着以参与式与公民主导的知识倡议来应对地下水治理改进挑战的关注度不断提升，社会—水文地质学有望揭示地下水与社会之间复杂的关系。尽管社会—水文地质学倡议在地下水研究者与实践者中日益受到重视，但其多嵌入实证主义、定量化的研究路径，并通常由水文地质学者主导，较少整合批判社会科学的思维。基于批判性水研究与社会水文学的最新成果，本文提出应对社会—水文地质学进行“情境化”，通过推进对印度西部Maharashtra地下水监测实践的跨学科理解，来推动这一研究取向的发展。本文以对地下水官员与农业社区地下水知识生产实践的定性研究为基础。研究发现表明，地下水监测的科学实践与替代性的、基于地方经验的地下水认知方式之间存在相互作用与融合。本文强调，相较于由国际、国家与区域机构推动的技术化、远距离监测形式，“在场的野外实践”具有重要价值。研究进一步揭示，地下水官员在日常监测工作中如何应对多重挑战，以实现“超越水文地质学”的实践取向——其中包括为避免官僚性负担而作出的若干“非正式”决策。最后，农业社区对地下水的理解深深嵌入其与地下水及农业之间的文化与物质关系之中，从而提供了替代性的地下水认知形式。总体而言，这些发现支持将社会—水文地质学情境化，推动其成为一种扎根于地方、以场所为基础的跨学科研究事业，从而为实现地下水可持续性的公正转型提供知识支撑。

## Resumo

Com uma ênfase crescente em iniciativas de conhecimento participativas e lideradas pelos cidadãos para enfrentar desafios na melhoria da governança das águas subterrâneas, a sóciohidrogeologia está bem posicionada para revelar as complexas relações entre a água subterrânea e a sociedade. Iniciativas sóciohidrogeológicas, embora ganhando força entre estudiosos e profissionais de águas subterrâneas, estão inseridas em abordagens positivistas e quantitativas, lideradas por hidrogeólogos, raramente integrando percepções das ciências sociais críticas. Baseando-se em trabalhos recentes em estudos críticos da água e sóciohidrologia, este artigo defende a 'situação' da sociohidrogeologia avançando uma compreensão transdisciplinar das práticas de monitoramento das águas subterrâneas no estado de Maharashtra, no oeste da Índia. O artigo baseia-se em uma investigação qualitativa sobre práticas de produção de conhecimento sobre água subterrânea com autoridades de água subterrânea e comunidades agrícolas. As descobertas do artigo demonstram a interação e integração entre as práticas científicas de monitoramento das águas subterrâneas e formas alternativas, baseadas no local, de conhecer a água subterrânea. Isso ressalta o valor de 'estar no campo' em detrimento das formas tecnológicas e distantes de monitoramento que estão sendo mobilizadas por agências internacionais, nacionais e regionais. Ele revela como os responsáveis pelas águas subterrâneas enfrentam desafios nas práticas cotidianas de monitoramento para ir 'além da hidrogeologia' – envolvendo decisões tomadas 'fora dos registros', moldadas por preocupações em evitar encargos burocráticos. Por fim, o entendimento sobre a água subterrânea em comunidades agrícolas está enraizado em suas relações culturais e materiais com a água subterrânea e a agricultura, oferecendo assim formas alternativas de conhecimento da água subterrânea. Coletivamente, essas descobertas avançam um argumento para situar a sóciohidrogeologia em um empreendimento transdisciplinar baseado no local, que promova mudanças justas para a sustentabilidade das águas subterrâneas.

## Introduction

The challenge of groundwater depletion is increasingly realised across the globe. Experiences from the North China Plain, midwestern United States, Australia, Spain, Yemen, Saudi Arabia, Syria and Chile suggest aquifers are in steep decline (Wada et al. 2010; Bessire 2022). Scholars are calling this a global groundwater crisis, as groundwater is now the most extracted natural resource in the world (Konikow and Kendy 2005, Famiglietti 2014). The situation is no different in the context of South Asia, where the Indus basin of Pakistan and India, the Indo-Gangetic plains of India and Bangladesh and hard rock regions of peninsular India are experiencing threats of groundwater depletion (Mukherjee 2018). This threat of depletion is alarming given that about 700 million Indians depend on groundwater for their drinking water needs, and 65% of irrigated agriculture in India is sourced through groundwater (Kulkarni et al. 2015). Groundwater has played a key role in alleviating poverty, improving access to irrigated agriculture, and in sustaining the drinking water security of millions of Indian households (Shah 2018; Pahuja et al. 2010). The climate crisis will further exacerbate the vulnerabilities of communities dependent on groundwater, with scholars pointing to a reduction of about 68% in cropping area in regions experiencing groundwater depletion (Jain et al. 2021) and drinking water scarcity (Saleth 2011).

Amongst the series of responses identified and mobilised to improve groundwater governance across the globe, there has been an emphasis on groundwater monitoring and measurements. Scholars, practitioners and international organisations posit that groundwater monitoring enables the establishment of the state of over-abstraction in an area or an aquifer, through observations and measurements of water levels that point to a series of actions involving the use of instruments and field observations (Varady et al. 2016; UNESCO 2022), while also outlining the importance of monitoring for groundwater quality (Ravenscroft and Lytton 2022). Groundwater monitoring involves “systematic measurement/observation and recording of the state of an aquifer, which is usually changing due to natural (e.g. change in precipitation pattern) and human (e.g. water abstraction) impacts” (UNESCO 2022). Many countries across the world have established monitoring mechanisms to produce groundwater knowledge to inform policies, programmes and governance responses (IGRAC 2020).

Although data and observations are key to sustainable groundwater management, groundwater monitoring presents challenges. Groundwater monitoring, unlike monitoring of other environmental phenomena or surface water systems, is challenging “due to hidden and three-dimensional nature of groundwater flow” (UNESCO 2022: 149). Unlike surface water that flows and accumulates in streams, springs, rivers and lakes that are ‘visible’ to a surface-level observer, groundwater is invisible (Alley et al. 2016). This is further complicated because of the diverse spatial and temporal scales of groundwater accumulation and movement (Schlager 2006). Groundwater recharge and residence times vary across aquifers and regions, thus challenging the standardisation of monitoring design and frequency. Within the Indian context, scholars and practitioners posit the emergence of a data-related issue stemming from a scarcity of monitoring stations, insufficient data collection frequency and adequacy, and accessibility or public disclosure issues pertaining to data gathered by governmental bodies (Chinnasamy and Agoramoorthy 2015; Kulkarni et al. 2015; Hora et al. 2019), a problem reported in other contexts as well (see Margat and Van der Gun 2013; UNESCO 2022).

Within the series of responses, socio-hydrogeological approaches can be one of the ways to engage and address the concerns. Building on the conceptual and practical applications of socio-hydrology that recognises the socio-natural character of water-society relationships (Sivapalan et al. 2012), and recognising the peculiar characteristics of groundwater resources, the field of socio-hydrogeology has evolved as a “sustainability science,” that engages with challenges of groundwater management and governance (Hynds et al. 2018). Scholars and practitioners of groundwater governance agree that socio-hydrogeology can play a vital role in integrating social dimensions in producing groundwater knowledge (Re 2015; Limaye 2017) and informing regional strategies (Aslekar et al. 2022). It has led to calls for building interdisciplinary approaches through increasing collaborations between hydrologists, hydrogeologists and social scientists (Rusca and Di Baldassarre 2019).

While socio-hydrogeology is a promising endeavour, building on the work of other scholars, this paper raises three concerns in current approaches to socio-hydrogeology. One, an examination of socio-approaches reveals how work by hydrologists and hydrogeologists is driven by positivist methodologies that promote neutral, apolitical epistemic framings for understanding water and groundwater (Wesselink et al. 2017). In doing so, they lead to pre-eminence of certain ways of knowing over others, what scholars call the techno-managerial approach, embedded in the hydrological discipline that focuses on the biophysical and managerial premises, thus evading “the reality of governance as a sociopolitical phenomenon” (Closas and Villholth 2020). Second, scholars argue that collaborations between natural and social scientists, expected in socio-hydro(geo)logical approaches, are affected due to asymmetrical integration between sciences that emerges through hierarchies of the sciences, leading to superiority of quantitative methods and relegating qualitative social science inputs as a plug-in (Rusca and Di Baldassarre 2019). This undermines the call for trans-disciplinarity in socio-hydrogeology, as the scholarship does not encompass the insightful work undertaken by social scientists that questions the epistemic framings and associated prescriptions for groundwater and water governance (Budds 2009). Third, the socio-hydrogeological work has attempted to identify global solutions that promote replicable models and frameworks (Hynds et al. 2018). In doing so, these approaches miss an opportunity to mobilise situated, contextual approaches to groundwater knowledge production and management that move beyond the business-as-usual approaches to groundwater governance.

This paper, through encounters with practices of groundwater monitoring in the western Indian state of Maharashtra, attempts to engage with these concerns by making a case for *situating* socio-hydrogeology. Drawing from critical water studies led by social scientists, this paper advances socio-hydrogeology to challenge the universal, politically neutral framings of groundwater knowledge that techno-managerial practices of groundwater monitoring and governance promote (Joy et al. 2014). Second, departing from quantitative framings of groundwater–society relationships, this work foregrounds qualitative inquiry that examines everyday practices of groundwater monitoring. In doing so, it attempts to disrupt the knowledge hierarchies between social and natural scientists, and between *expert* hydrogeologists and local communities while identifying new pathways for knowledge to travel (Zwarteveen et al. 2021). Third, moving away from global framing of socio-hydrogeology as a “replicable solution” (Re 2015), that relies on *objective* and *neutral* scientists, officials and technologies, it foregrounds the value of human agency and socially situated practices of groundwater knowledge production to enrich socio-hydrogeology’s strength of being grounded and place-based transdisciplinary enterprise.

*Situating* socio-hydrogeology for groundwater monitoring and governance becomes important for three reasons. One, the increasing role of participatory approaches to groundwater governance through programmes that emphasise the role of community in evolving management strategies (Kulkarni et al. 2015, Closas and Vilholth 2020). Second, the calls for improving and integrating citizen science, including a call by UNESCO’s Intergovernmental Hydrological Programme (IHP), in solving the challenges of data and information in water and groundwater governance across different parts of the globe (UNESCO 2022). This also links to efforts that adopt co-production as an epistemic practice for groundwater knowledge production (Baldwin et al. 2012). Last, the arena of groundwater and water governance is informed by concern of justice that moves beyond distributional and social justice to integrate questions about epistemic and knowledge justice (Haeffner et al. 2021). This paper contributes to these pertinent discussions and scholarship by exploring the socio-hydrogeology of groundwater knowledge production with an emphasis on practices of monitoring.

The paper is organised as follows. The following *Situating socio-hydrogeology* section explores a role of situated socio-hydrogeology in practices of groundwater monitoring and how it may help address challenges of groundwater governance. Methodological considerations, including field site and data collection methods, are discussed in the *Field area and research methods* section of the paper. The *Groundwater monitoring in Maharashtra, India* section offers background information on groundwater monitoring within Maharashtra, India, followed by the *Situating groundwater monitoring* section (and *Of hydrogeologists and water diviners*, *Moving beyond the water level: Value of fieldwork and observations*, *Beyond hydrogeology: Everyday practices of monitoring* and *Situating groundwater observations through cultural and material relationships* sub-sections) that details the key findings emerging from an examination of groundwater monitoring practices in the state. The *Discussion* section discusses the relevance of these findings within the broader debates on socio-hydrogeology and suggests their applications for improving how groundwater comes to be known, followed by concluding remarks in the *Conclusions* section.

## Situating socio-hydrogeology

The invisible nature of groundwater and the associated properties of being horizontal (across the area of an aquifer) and vertical (depth of aquifer) make a case for a distinction from socio-hydrology to socio-hydrogeology. This is embedded in the disciplinary distinctions of hydrology and hydrogeology and the nature of surface water (loosely, water) and groundwater governance (Molle and Closas 2020). Unlike surface water, groundwater is deemed a horizontal, ubiquitous resource in programmes and policies that enables decentralised access across the landscape (Walsh 2022). This shapes a distinct form of knowledge systems that is decentralised, networked and scattered in the form of hundreds of monitoring points. Another distinction from surface water systems stems from the element of depth, of verticality in the nature of aquifers: groundwater availability (or lack thereof) varies across the aquifer and with depth. Together, these characteristics of groundwater bring into focus a distinct set of practices and processes associated with producing groundwater knowledge that can be informed through a *situated* socio-hydrogeological approach, as elaborated in this section.

In line with the wider groundwater scholarship, the paper emphasises the need to focus on socio-hydrogeology to effectively address the challenges between groundwater and society for the following reasons. Socio-hydrogeology is deemed to be a transdisciplinary endeavour that seeks to bring together insights from different disciplines to address the challenges of groundwater governance (Hynds et al. 2018). As outlined above, groundwater monitoring is complex and challenging, and socio-hydrogeology can prove to be a valuable approach through the integration of social dimensions into hydrogeological investigations (Re 2015) through its emphasis on the role of hydrogeologists in the field, fostering dialogue with communities (Limaye 2017). In doing so, they suggest hydrogeologists *become* ‘social hydrogeologists,’ in that they engage with farming communities to ask questions about groundwater use, access, cropping choices and ownership of lands that inform their mapping and monitoring processes. Lastly, identified as a ‘sustainability science’ (Hynds et al. 2018), socio-hydrogeology has a solution-oriented approach that attempts to address the challenges of groundwater management and governance. This approach posits the role and value of socio-hydrogeology in groundwater policy and practice to foster its integration in programmes.

However, as outlined in the introduction, mobilising socio-hydrogeology as a politically neutral, replicable (universal) approach reinforces disciplinary hierarchies that derail the opportunity of developing a grounded, transdisciplinary approach for addressing groundwater–society challenges. This paper calls for drawing on insights emerging from critical water scholarship, led by social scientists and critical physical geographers, to engage and address these concerns. Scholars argue that bringing together socio-hydrology (and thereby, socio-hydrogeology) and critical water scholarship can suggest ways to improve and integrate concerns of justice and participation in knowledge production practices and processes (Rusca and Di Baldassare 2019). They agree that a deeper engagement with the social sciences is crucial to integrate new methods and perspectives into socio-hydrological approaches (Seidl and Barthel 2017; Xu et al. 2018). While this discussion can lead in many directions to encompass other facets of the socio-hydrogeological approach (develop management plans/ strategies, shape programmes and policies), the focus of this paper is on the practices of groundwater knowledge production, particularly groundwater monitoring (with a focus on quantities than qualities of groundwater). Subsequent paragraphs discuss insights emerging from this scholarship that are engaged through this paper to further a case for *situating* socio-hydrogeology.

The socio-hydrogeological endeavour attempts to bring together the social and hydrological by *embedding* the social dimensions in hydrogeological investigations (Re 2015). In doing so, it posits that the social is delineated from the hydrological and hence needs to be *embedded*. This understanding of the ontological separation between nature and society shapes practices of knowledge production and how groundwater comes to be known. It leads to pre-eminence of hydrological disciplines that attend to the biophysical aspects of water/groundwater, reinforcing knowledge hierarchies between local and expert, scientific and non-scientific. To *situate* is to acknowledge that all knowledge practices are *situated* and are based on specific experiences (Haraway 2004; Zwarteveen et al. 2021). Haraway (2004) questions the techno-scientific gaze from nowhere, troubling the separation between nature and culture, suggesting that scientific practices (of knowledge production) are not solely technical and neutral but shaped by both scientific representations of nature and scientists’ research cultures and interests (Demeritt 2001). As this paper engages with the question of groundwater monitoring practices that inform how groundwater comes to be known, the concept of *situated* knowledge enables critical examination of the practices to question their neutral, universal framings.

Work by critical water scholars has revealed how hydrological and hydrogeological practices (and thus socio-hydrogeology) are shaped by socio-cultural factors. Drawing on science and technology studies (STS) the work suggests how “science as practice….is not necessarily the same as science as it is portrayed” (Lane 2014). Practicing hydrogeology involves dealing with uncertainties and other constraints that require judgement, which are often *situated* within broader social contexts and conditions (see Condon et al. 2020). As Tadaki et al. (2015) observe about practices of environmental scientists (in this case, hydrogeologists): “it is about ‘representing the real’ of something from somewhere, and perhaps by someone with a specific set of theoretical underpinnings and values” (2015). Stuart Lane, in his analysis of socio-hydrological work, questions the separation between scientists and society. According to Lane, “hydrological knowledge … cannot be understood if it is divorced from the networks within which it is produced, that is, an assemblage of elements that are material (e.g. conservation of fluid mass, flood defences), technical (e.g. state of knowledge, computational power), regulatory (e.g. defined modelling procedures) and human (e.g. ability to improvise, perception)” (Lane 2014).

Positing socio-hydrogeology as an approach for “taking science to society” (Limaye 2017) exposes a hierarchy of knowledge and reinforces the strict binaries of scientific and non-scientific, local and expert. In their work in Tamil Nadu, India, Verzijl et al. (2023) reveal how groundwater modellers draw and build on conceptualisations put forth by water diviners, thus arguing for the value of knowledge deemed ‘beyond scientific’. Water diviners are primarily local people, with expertise in locating underground water through traditional methods usually employing divining tools like coconuts, copper wires or other materials. Through their practice, water diviner “seeks to improve farmer livelihoods, gets compensated, and senses the right place for a well (which is not about whether you could, but also whether you should dig at a certain location)” (Verzijl et al. 2023). The authors, through careful, symmetrical analysis, argue that both practices—modelling and divining rely on set of logics that are celebrated within specific networks (Mol 2002), and hence instead of being examined hierarchically, could “accompany each other to address sustainability and justice concerns in groundwater governance” (Verzijl et al. 2023).

As groundwater issues become increasingly challenging, hydrogeologists and physical geographers propose new forms of monitoring groundwater through automated sensors, telemetry, and remote sensing imagery (Dwivedi et al. 2024; Rodell et al. 2009). In India too, through World Bank funded successive National Hydrology Projects, there is a massive push towards remote, and technology assisted groundwater monitoring mechanisms that enables agencies to monitor water levels through Digital and Automated Water Level Recorders (DWLRs & AWLRs) that record and relay data on a set frequency to the office or data centre (World Bank 2018). While part of these developments stems from the need for the state to make groundwater legible (Birkenholtz 2015), it is recognised as being impartial and reliable in contrast to manual forms of monitoring (Varady et al. 2016).

But does the removal of humans from the site make monitoring objective and neutral? In her work on the national program for mapping groundwater resources in Costa Rica using remote sensing technologies, Ballestero (2019) observes how such technologies often promote objective and modernist assumptions of ‘seeing’ things; and drawing from Haraway (2004) argues that seeing (graphs, maps and models) is not a neutral activity, it is a “cultivated, theoretically and culturally laden practice” (Ballestero 2019). While one may point to remote and automated monitoring methods as ‘objective’, they involve decisions about where a piezometer comes up, thus prompting questions of who decides, what concerns shape those decisions. This paper, by elucidating the choices and subjectivities involved in both ‘expert’ and ‘automated’ monitoring, opens the possibility of exploring the epistemic politics of considering and valuing human agency.

## Field area and research methods

### Study location

The findings of this paper come from ethnographic work carried out in the Marathwada region, within the Osmanabad (Dharashiv) district of the western Indian state of Maharashtra, that has been at the forefront of discussions of water scarcity, droughts and groundwater depletion (Purandare 2013). As this work attends to the practices of groundwater knowledge production, which are often characterised as a technical predicament, an ethnographic approach offers a situated, open-ended, qualitative route to examine them (Bessire 2022). Thus, instead of practices as outlined in guidelines and hence impartial and neutral, this paper approaches practices of groundwater monitoring “as always in-the-making and inherently performative.” (Kemerink-Seyoum et al. 2019).

Based on prior experience of working in the region, a micro-watershed, part of MR36A (MR describes that watershed is part of Manjara river basin) watershed in the Osmanabad district (sub-administrative unit of Maharashtra state), was identified as the primary field site for two reasons: One, the set of five villages that constituted the micro-watershed were identified as project villages under Atal Bhujal Yojana by national and regional groundwater agencies. Atal Bhujal Yojana (ABhY) is the flagship programme for participatory groundwater management led by the national government of India with support from the World Bank that emphasises an improved understanding of groundwater resources (World Bank 2018; MoJS 2020). Under the programme, piezometers equipped with digital water level recorders (DWLRs) must be established in each project village to ensure continuous water level data recording and “to allow for accurate measurements of localised impacts of program” (World Bank 2018). This gave an opportunity to explore practices associated with setting up and undertaking groundwater monitoring in the villages. Second, watersheds are the unit of groundwater knowledge production in the state of Maharashtra. Thus, identifying a watershed as the field site enabled exploring practices of monitoring, visiting observation wells, examining documents and guidelines that helped inform questions for interviews. Beyond the primary field site, fieldwork was also conducted at district and state offices of the state groundwater agency- Groundwater Surveys and Development Agency (henceforth, GSDA) to interview officials. The library at the Directorate office of GSDA at Pune was valuable for accessing organisational documents, including reports, manuals, and government circulars.

### Research methods

A set of three interrelated qualitative research methods were deployed for data collection: a) semi-structured interviews, b) walking (taking a traverse) and c) document analysis. A total of 19 semi-structured interviews were conducted with state groundwater officials, retired officials, regional, national and international consultants, practitioners from non-government organisations (NGOs) and farming communities of the MR36A watershed (see Table 1). While the ABhY project documents and agency-issued guidelines formed the basis of leading questions for the officials, preliminary discussions and observations in the villages formed the basis of semi-structured interview questions for farmers from the five villages. The intention behind this process was to “uncover and describe the participants’ perspectives on events – that is, the subjective view is what matters” (Marshall and Rossman 2014). Interviews with officials ensured representation across organisational hierarchy (Senior Geologist, Junior Geologist), gender and location (State Directorate Office, District office) while in case of farmers, interviews were conducted with farmers from different caste groups (Dhangar, Maratha) and landholding (small and large). Some independent consultants and practitioners were also interviewed, identified through leads emerging from discussions and prior knowledge about their work. Interviews were conducted in the Marathi language, recorded in the field using audio recording devices, followed by field notes written at the end of the day. The interviews were later transcribed and translated into English for the purpose of analysis. Analytical themes were identified from interviews through an inductive, iterative process. All interview participants described in the paper are anonymised to protect their identities and address ethical concerns.

**Table 1:** Total number of interviews across different research participant groups

Nature of participants	Total number of interviews
Farmers	8
Groundwater agency officials	7
Consultants (non-affiliated, independent, retired)	4

In the case of water studies, walking through a landscape, through a watershed or a river basin may enable narratives, knowledge and non-human representations which otherwise may be missed or neglected in more stagnant/static methods of data collection. Holstead et al. (2025) posit walking as “practice based method embedded (in the landscape and routines) and embodied (using the body, mind, and senses) to discover, open up, and represent relationships to water.” In case of this paper, the method of walking was also inspired from hydrogeologists (geologists), who use the method of “conducting a traverse” (Compton 1986), through a landscape with aid of a base map like a toposheet and a compass, identifying a path (a predetermined route) and then traversing or walking (or sometimes driving if the distance to be covered is extensive, basically moving) along the path to collect geological data pertaining to rock types, samples of rocks, and records changing rock strata, dug-wells, groundwater sources, other physiographic features like streams and topographic variations. To reveal and unpack the cultural and material relationships with groundwater, interviews with farmers followed the method of walking on farms, led by them, near dug-wells and other forms of water infrastructures while talking about their practices and experiences that inform ways of knowing groundwater. Detailed notes about these walks were made later, and audio interviews with farmers were recorded along with photographs wherever deemed necessary.

Document analysis was undertaken for project reports produced by GSDA, World Bank and other national and international agencies. Analysis of institutional guidelines, manuals, norms, groundwater assessment reports, and research articles by groundwater agency officials was undertaken to identify practices and processes of groundwater monitoring. Document analysis entailed “finding, selecting, appraising (making sense of), and synthesising data contained in documents” (Bowen 2009), which provided historical insights, background information to inform and contextualise data collected from other methods. Insights emerging from this analysis formed the basis for identifying leading questions in interviews with officials.

## Groundwater monitoring in Maharashtra, India

According to the Government of Maharashtra, groundwater exploitation has reached unsustainable levels in the state (GSDA 2014) and based on recent assessment about 23% of the blocks (administrative unit part of district) in the state have been identified as semi-critical, critical or overexploited (CGWB 2020). Over 2.1 million irrigation wells dot the state; rural water supplies rely primarily on groundwater schemes (GSDA 2014, Sakthivel et al. 2015). Given this context, governing and managing groundwater demands the state’s immediate attention, impacting livelihoods, drinking water security, and irrigation. The state has a long history of groundwater monitoring and groundwater resource assessments undertaken by the state’s groundwater department, GSDA.

“It may be stated that like the pulse in the human body, the behaviour of water levels forms a valuable diagnostic tool in the hands of the Hydrogeologist to understand the health of groundwater both on the regional and micro (watershed) levels.” (Maggirwar & Umrikar 2011)

The above quote, from a paper by GSDA officials, points to the importance of ‘water levels’ in understanding groundwater. It outlines the relevance of monitoring water levels or its ‘behaviour’ to understand the ‘health’ or the state of groundwater resources in a particular region. According to GSDA, every region, area, village, watershed, or aquifer has a certain ‘groundwater potential’, and an understanding of this potential can be established through groundwater assessments derived based on monitoring of water levels, which is the key idea behind establishing monitoring observation wells (GSDA 2014). Agencies refer to it as a ‘network’ because water levels are monitored at multiple locations throughout the state (CGWB 2020). Together, they provide a comprehensive picture of the state of the groundwater resources in a district, taluka, and watershed level.

GSDA subscribes to a methodological framework of groundwater monitoring that involves identification of ‘ideal monitoring wells’, establishing a frequency of monitoring water levels throughout the year (usually four times) and using a specific set of formulas to undertake estimations or assessments of groundwater resources in the state (Maggirwar and Umrikar 2011). Currently, 3905 such open-dug wells constitute the network of monitoring in the state, with at least one well every 80 km^2^ (GSDA 2014). New observation wells and telemetric monitoring stations, implemented by the World Bank-supported National Hydrology Programme (NHP), or piezometers installed as part of projects like Atal Bhujal Yojana (ABhY) are further enhancing the monitoring network (World Bank 2018). The state has also seen a shift from agency-led to community-led, participatory forms of groundwater monitoring under the innovative groundwater governance programmes implemented with support from international agencies like the World Bank (Argade and Narayanan 2019).

While monitoring typically relies on aquifer boundaries (IGRAC 2020), in India, monitoring is based on administrative units to align with policies and programs, or watersheds in the case of hard rock regions (CGWB 2020). Maharashtra’s groundwater monitoring network is based on watersheds to produce groundwater resource estimation reports that are further apportioned to an administrative unit like a block and district to support the implementation of programmes and policies (GSDA 2014).

## Situating groundwater monitoring

This section, through an attention to practices of groundwater monitoring, outlines the four empirical themes that advance the case for situating socio-hydrogeology. First, it begins with outlining how diverse knowledges, those of hydrogeologists and water diviners, shape groundwater monitoring practices. Second, it reveals how manual (on-site) and remote monitoring practices are both situated—it matters who makes the visits, who observes the data on the graph underlining the value of human agency in an objective, universal science of hydrogeology. The third subsection points how officials move beyond hydrogeology to integrate other concerns while undertaking everyday practices of monitoring. Lastly, moving away from the work of agency and officials, the section illustrates how farming communities, through their cultural and material relationships with groundwater and agriculture, come to know groundwater.

### Of hydrogeologists and water diviners

State groundwater agency guidelines (GSDA) define an ‘ideal monitoring well’ for tracking groundwater level fluctuations. To maintain accurate water level measurements, the well should be an ‘open dug-well’, representative of homogenous local conditions and non-pumping for irrigation, to avoid influence from nearby wells (Maggirwar and Umrikar 2011). The observation well should represent the aquifer such that it should be ‘covering full aquifer thickness’, indicating that such a well should be an independent source of representation for the aquifer. These set of criteria, coupled with a limited programme budget to develop new monitoring wells in the 1970s, led to the identification of Aad, a common type of dug-well for groundwater monitoring purposes by GSDA in many parts of the Marathwada region of the state (interview SM 8 June 2022, interview JP 03 Jan 2024). In most parts of the region, these ‘puratan’ (traditional) sources of groundwater continue to be deployed for groundwater monitoring (field notes 27 March 2022).

**Fig. 1:**
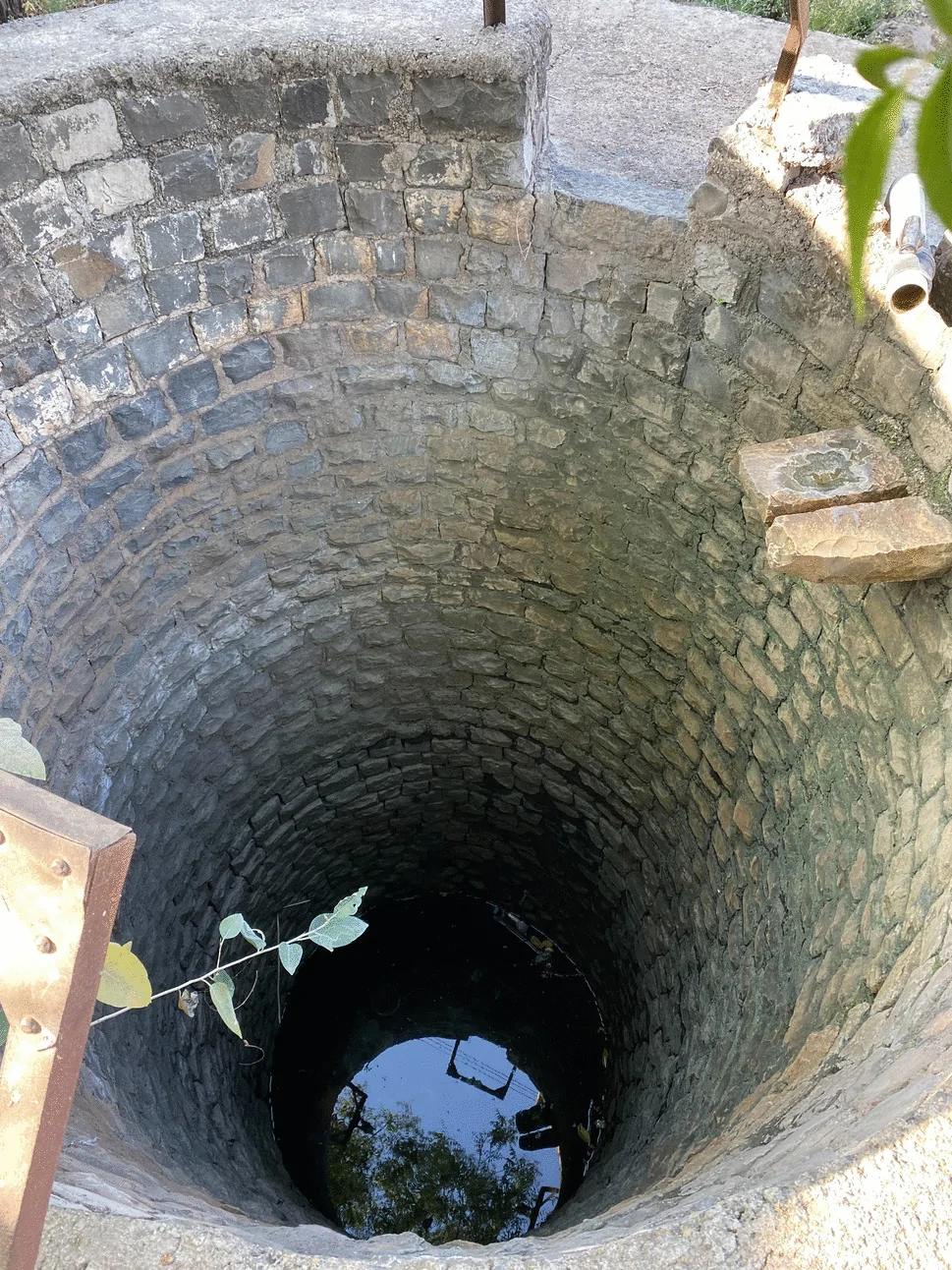
An Aad in Harali village. Photograph accreditation: Dhaval Joshi

Aad are traditional dug-wells used as drinking water sources that do not have pumps installed on them and water is hand-drawn by community members (see Fig. 1). Relative to irrigation dug-wells, Aad are small diameter (ranging 2–5 m), deeper dug-wells. They are deep enough to represent the shallow subsurface and are often found in village habitations, making it easier for communities to fetch water. Their location in habitations prevents any interference from the pumping of irrigation wells. Most of these were constructed during the administration of the Nizam of Hyderabad in the pre-independence era (before 1947). The locations of these Aad, or as is the case with most groundwater sources, constructed in the villages, were derived with help from water diviners, locally referred to as ‘panadya’ (field notes 17 Feb 2022 and 03 July 2022). In the field site, these water diviners were mostly men, belonging to the local community or from nearby villages, who were recognised by their ‘success rate’ in helping farmers locate underground water sources. Except for drinking water supply schemes wherein groundwater agency officials are involved in suggesting the site or location of the dug-well, most sources are derived with the help from water diviners (interview DP 15 May 2022). In many villages, Aad have been classified based on caste like Maratha Aad, Dalit vasti Aad to determine which caste groups can access them. Maratha caste communities are dominant agricultural communities in rural Maharashtra, while Dalits, recognised as Scheduled Castes under the Indian constitution, are one of the most oppressed caste groups that face discrimination from dominant caste groups. The segregation of water sources, in this case Aad, based on caste, reveals existing social inequities that shape knowledge and access to water (see O’Reilly and Dhanju 2014).

Using Aad for groundwater monitoring by the state groundwater agency reveals how knowledge is hybridised, highlighting how boundaries of scientific and non-scientific are blurred in identifying the ‘ideal monitoring well.’ It points towards approval or legitimisation of other forms of groundwater knowledge, the term ‘other’ implying one that is maintained and practiced by many water diviners in the region, emerging through distinct set of practices. While both the hydrogeologist and the water diviner, ‘map’ the area for groundwater, they rely on distinct theoretical framings about the subsurface and employ distinct methods to explore groundwater in the subsurface. Their entanglements or assimilation in ‘scientific knowledge-making’ practices of monitoring wells points to the “intimately entangled” (Verzijl et al. 2023) nature of knowledge, one that builds, relies, and draws upon these Aad.

Following this exploration of invisible entanglements, the next section will dwell to discuss how officials value fieldwork and how field-based observations and understandings build into the measurement of water levels while also examining whether remote monitoring practices deemed impartial and hence reliable, are devoid of human agency.

### Moving beyond the water level: Value of fieldwork and observations

“One medicine does not fit all; see the situation of the Kalamb block. If you ask me whether the water level shown in the reports can be observed in that area, I will deny that as I have practical experience.” (Interview SM 08 June 2022)

The above comment by Osmanabad district groundwater official Sukanya Mane, with extensive experience of working in Osmanabad and adjoining districts, outlines the discomfort of the gap between what is being reported and what appears to be a ‘practical experience.’ While water level data seen on graphs or records from the area depicts a way to make water level legible in a ‘report’, she did not value this over what is ‘visible’ through her practical experience. The disparity between what’s observed directly and what’s recorded in files or graphs is revealed through Sukanya Mane’s perspective. On the question pertaining to this disparity, Usha Shinde, an official working with the Osmanabad district GSDA office, shared:

“A geologist, unlike a geophysicist, undertakes groundwater surveys by naked eye.” (Interview US 10 June 2022)

Usha Shinde and Sukanya Mane’s comments point to the value of ‘being there’ and ‘doing fieldwork’, an approach reiterated repeatedly through manuals and guidelines produced by the GSDA over the decades. In the guidelines issued under a report titled ‘Instructions for systematic hydrogeological survey’ outlined by the department in the 1980s:

“…one must be familiar with regular geological mapping and convert his skill to identify dug-wells as the units of observations. The geological data encountered in dug-wells will help to understand the groundwater regime in its all perspective in the given area under survey work.” (GSDA undated)

The paragraph above explains the value of the dug wells as units of observation. This highlights the importance of converting one’s skills to identify wells for groundwater-related observations associated with local geology and water levels. Furthermore, the report outlines the value of note-taking and field notes:

“…field notes are always taken in the field on the field observations and impressions. Note taking should not be substituted by depending on one’s memory and writing them in camp.” (GSDA undated)

For a department that values fieldwork, the introduction of remote monitoring practices presents a new set of challenges. According to John Palaniswami, a former GSDA official, the development of AWLRs comes with a ‘dis-advantage:’

“Today’s boys what they are taking data (of groundwater), they do not know field conditions. When we used to bring it (water level readings) manually, you would walk, you would see what the land use pattern is, what is cropping pattern, right, what is soil, geology, we used to see all this. What happens with DWLR? He sees the well on the graph. He does not know where the well is (located). He does not associate with that well. In hydrogeology, you cannot do that.” (Interview JP 03 Jan 2024)

In his opinion, such field observations are important for groundwater assessments. He recollected how senior officials would entrust him with data organisation and analysis to develop groundwater reports, because of his extensive field engagement. He shared that the official visiting the field:

*“visualises the well* (across different seasons), so those who were very good in the field used to enter data in the GEC (methodology), why because every data is not documented.” (Interview JP 03 Jan 2024, emphasis added)

These comments illustrate that remote monitoring practices are also *situated*- who observes—thus challenging groundwater monitoring practice as objective and universal. It reveals that the practice of groundwater monitoring is beyond the simple act of measuring water levels by reiterating the value of fieldwork and being good at doing it as an important precursor for handling/managing remote data for assessments. It foregrounds the importance of who performs these field observations, and in this case, *inverts* the hierarchy of officials. Most importantly, it situates the hydrogeologists or the groundwater official in this case, in the field, thus enabling their specific experiences to emerge through discussions, observations, and exchanges with communities that depend on groundwater for everyday needs. The next section delves into how officials navigate the challenges of undertaking groundwater monitoring and associated decisions, and move beyond the guidelines and norms set by the department.

### Beyond hydrogeology: Everyday practices of monitoring

While norms, guidelines and prescriptions define the roles and responsibilities of groundwater officials in undertaking their work, an attention to everyday practices reveals decisions that are ‘off the record’ and shaped by pragmatics of funding, project timelines, and avoiding bureaucratic burdens. By exploring the decisions about sites of monitoring, this section unpacks how they are shaped by concerns ‘beyond hydrogeology,’ moving away from guidelines, norms and protocols identified and established by the GSDA.

While visiting the field site, a senior GSDA official from the Osmanabad district office planned to survey the village to identify a suitable location for the monitoring well- a piezometer- under ABhY programme. While beginning the survey, the official suggested that one of the most important criteria for identifying the location is that it should be located on a ‘sarkari jamin’ — i.e., government land (field notes 11 February 2022). This can be on the premises of the Gram Panchayat, the school, or any other institutional or government-owned land. In an interview with a senior GSDA official, Deepa Gokhale, at the state office of the agency (Directorate), with experience of working on various World Bank-supported programmes, these are ‘off the record’ decisions- deviating away from guidelines/norms and hence not included in project reports- enabling officials to navigate the challenges of implementing project activities. She shared:

“What happens off the record? If we do it (install piezometers) anywhere, we see it is destroyed, vandalised. So, off the record, we construct monitoring piezometers in school’s premises, Gram Panchayat office and other such places to avoid such issues.” (Interview DG 14 Jan 2022)

Similar decisions were found in other activities, such as identifying a drinking water source, wherein officials, to prevent legal and other bureaucratic burdens, identify sites that would be legally safe, moving beyond hydrogeological observations to integrate other concerns and prevent any potential conflicts in the future (Interview SM dated 07 June 2022). In the adjoining village, the district project unit working with GSDA had installed the rain gauge on the roof of the Gram Panchayat building, the village council office. Doing so may have flouted the norms for accurate measurements of rainfall but ensured that the rain-gauge is installed away from potential harm, while ensuring that the rain-gauge was installed on ‘sarkari jamin’ (government-owned land). These observations about identifying ‘sarkari jamin’ as sites for piezometers (and rain-gauge) reveal the decisions ‘beyond hydrogeology’ in monitoring practices, as these are not identified for their hydrogeological relevance but to overcome bureaucratic concerns. While the GSDA’s discourse values a ‘scientific approach’ and gives legitimacy to it through training manuals and guidelines, the practices suggest that everyday decisions can move beyond the ‘scientific’ to include other ‘non-hydrogeological’ concerns. It underscores the significance of appreciating that hydrogeologists may not always be able to meet guidelines or maintain scientific norms in their fieldwork. While this and the earlier sections discussed the practices of groundwater monitoring by state officials, the next section outlines how farming communities come to understand groundwater fluctuations in their area, through cultural and material relationships with groundwater in the area.

### Situating groundwater observations through cultural and material relationships

The state agency’s methodology of understanding groundwater levels revolves around a fixed calendar. The agency officials record groundwater levels four times during the year, i.e. June, September/October, January and March (GSDA 2014). These recordings are then used in groundwater assessment formulas to produce an understanding of groundwater estimates in an area, region, or state. Contrary to this state’s methodology of ‘knowing groundwater’, interviews and discussions with farmers revealed observations associated with groundwater in the villages revolved around cultural and material relationships with farms and farming. In this section, I discuss these two aspects. First, cultural events inform understanding and references to groundwater conditions in the villages, particularly those occurring during the most critical phase of groundwater dependency, i.e. during the cultivation of post-monsoon or Rabi season crops. Two key post-monsoon cultural events were identified: ‘Vel Amavasya’ and ‘Padwa’. Vel Amavasya and Padwa occur during the winter and spring seasons, respectively, and featured prominently in interviews with farmers and in wider discussions and observation in the village. Second, interviews with farmers revealed how material practices related to farming like irrigation needs, crop water requirements, and groundwater pumping capacities and duration informed their understanding of groundwater, revealing a different relationship with groundwater.

Vel Amavasya is a farm-related festival wherein a ‘pooja’ (ritual involved in most Hindu religious practices that involves worship of a deity/deities) is undertaken on the farm about two to three months after sowing Rabi or winter crops. The festival is more prevalent in the southern districts of Maharashtra state, those bordering Karnataka state, where the festival is celebrated enthusiastically. The Padwa is also known as ‘Gudi Padwa’, which coincides with the spring season and is celebrated by most Hindu families. Unlike Vel Amavasya, Padwa is not an agricultural or farm-related festival. While Vel Amavasya usually occurs during December-January, Padwa is celebrated in March or April. These cultural events were brought up during interviews as points of reference by farmers while discussing the behaviour of their wells, or the water level fluctuations in them. Farmers, for instance, mentioned that their well water levels do not recover as much after pumping following Vel Amavasya, and that pumping groundwater becomes more difficult after Padwa, indicating that there is insufficient water for the pump to operate effectively (Interview with SKamble 18 March 2022, SKarnure, 04 July 2022). Likewise, the water-pumping capacity of wells during Padwa served as an indicator of a successful monsoon and favourable groundwater conditions in the village from the prior year. This reflects how cultural events serve as points of reference for knowing groundwater.

While cultural events were used as reference points to discuss groundwater conditions, it was the material practices of farmers that shaped their understanding of groundwater. Interviews revealed how well behaviour and groundwater conditions were associated with their capacity to pump water, irrigation water requirements and duration of pumping possible (Interview MK 13 May 2022; DP 15 May 2022, SW 7 March 2022). Farmers engage with groundwater sources for their irrigation needs and except in cases where annual crops like sugarcane are cultivated, farmers are more sensitive to water level fluctuations given the dynamic crop water requirements that shift throughout the growth period of the crops.

These interviews illustrated little or no reference to dug-wells and groundwater conditions during the summer or pre-monsoon period, suggesting that the material relationship with groundwater ceases to exist during those periods, as agricultural activity is negligible and communities await the beginning of the monsoon. That ‘pre-monsoon’ is rarely referred in local discussions on groundwater may lead to question observation about dry wells as recorded by the state agency (see Hora et al. 2019), and what this means for the groundwater estimation methodology for categorising the area in terms of ‘stage of groundwater development’. This section reveals how farming communities rely on cultural and material relationships with groundwater and agriculture to shape their understanding of groundwater in the area.

## Discussion

This paper initiated the argument that socio-hydrogeological approaches, while promoted to address groundwater knowledge and governance challenges, are undermined by their mobilisation as being politically neutral, objective and universal. More importantly, while being promoted as a transdisciplinary endeavour, socio-approaches have not addressed the knowledge hierarchies prevalent amongst hydrogeologists and social scientists. Drawing on critical water scholarship and work on the politics of environmental knowledge, the observations described in this paper advance the efforts to *situate* socio-hydrogeology through a transdisciplinary understanding of practices of groundwater monitoring. It does so by 1) recognising how diverse knowledge informs practices of groundwater knowledge production, 2) by valuing the fieldwork undertaken by hydrogeologists, groundwater officials and how discussions and observations can transcend a simple measurement of water levels, 3) by listening to and observing challenges faced by officials in everyday practices that move beyond hydrogeology and 4) moving away from practices of officials to *situate* observations of groundwater level fluctuations in socio-cultural contexts. This section weaves these observations within broader academic discussions with implications for practices of groundwater knowledge production.

Socio-hydrogeology is often identified as an approach that enables ‘taking science to society’ (Limaye 2015), by demystifying science and scientists to society (Re 2015). In doing so, it posits a hierarchy of knowledge, who produces it, and where it is produced. However, as revealed in this paper, local knowledge, deemed unscientific, can inform hydrogeological practices of identifying monitoring wells or informing groundwater conditions. The identification of Aad as ‘ideal monitoring wells’ illustrates how state-led practices of groundwater monitoring build upon other knowledges, in this case that of water diviners, thus blurring of boundaries between what is deemed ‘scientific’ and ‘non-scientific.’ It shows how formal hydrogeological knowledge is situated and contextual and, in doing so, questions the hierarchy of government/state knowledge over farmers’ knowledge. Tracing work of groundwater modellers and water diviners in Tamil Nadu, India, Verzijl et. al. (2023) shows that ‘beyond scientific’ techniques of water diviners and that of groundwater modellers both rely on local or in situ environmental information, mobilising different techniques “to forecast where and how groundwater flows but do so through different ways of knowing that are invisibly but intimately entangled” (2023). By acknowledging these varied knowledge sources and their importance in understanding groundwater, hydrogeologists working together with social scientists in transdisciplinary approaches, can help strengthen a case for situating socio-hydrogeology.

Monitoring practices have seen a shift from manual forms of monitoring towards automated, technology-enabled water level recording methods with the assumption that they are impartial and hence more reliable (Varady et al. 2016). Human involvement is central to manual measurements, such as when someone sees a measuring tape interacting with water in a well; conversely, automated measurements transfer this role to a sensor, a technological tool that gauges the water level. The findings of this paper challenge two premises about groundwater monitoring: that manual monitoring is just about measuring water levels and recording a numerical value, and that remote monitoring eliminates human involvement. Field visits, according to officials, help them observe the local environment, discuss farming practices with farmers, and gain a more comprehensive understanding of groundwater conditions beyond water level readings. Officials with such field expertise are better equipped to interpret and analyse remote data from graphs. Both findings emphasise the importance of ‘seeing’ as a cultivated, laden practice (Ballestero 2019), that moves away from a techno-scientific gaze from nowhere to positioning the official in the field and office. It highlights *who* makes the field visits, *who* is involved in reading and analysing collected data, thus placing an onus on human agency in both manual and remote monitoring practices. Thus, unlike as suggested in socio-hydrogeological approaches, hydrogeologists do not *act* as social-hydrologists (Re 2015) but are ‘social-hydrologists.’

Critical water scholarship on socio-approaches calls for hydrological/hydrogeological practices that are attuned to socio-cultural relations in knowledge production. However, as socio-approaches continue to be led predominantly by hydrologists and hydrogeologists, they call for *embedding*, incorporating social dimensions in hydrogeological investigations, especially in practices of groundwater knowledge production (Re 2015, Pande and Sivapalan 2017). Such an approach emerges from the ontological separation of nature-society that reduces the role of humans and society to social variables and indicators (Linton 2022, Zwarteveen et al. 2021). However, as revealed through different findings of this paper, hydrogeological practices are inherently *socially situated*. From practices of manual and remote groundwater monitoring to the beyond hydrogeological concerns that shape the monitoring decisions, the paper has highlighted how hydrogeologists, groundwater officials emphasise the value of fieldwork, while undertaking everyday practices of monitoring to navigate bureaucratic burdens. It questions the claims of objectivity and impartiality often promoted by techno-managerial framings of water/groundwater knowledge production practices.

While being promoted as a transdisciplinary endeavour, it is argued that socio-approaches do not incorporate the critical insights produced by social scientists (Xu et al. 2018). They observe an inclination for quantification, valuing qualitative inquiry as a plug-in**.** By foregrounding insights through qualitative inquiry, the findings of this paper facilitate an opportunity towards transdisciplinary approaches to understanding and addressing challenges of groundwater monitoring. By examining practices, which are often seen as strictly technical, and thus universal, this paper demonstrates how qualitative examination, coupled with insights from critical water studies led by social scientists, assists us in revealing their place-based nature in making groundwater knowable. Transdisciplinary work, unlike interdisciplinarity, transcends and integrates disciplinary paradigms in order to address socially relevant issues, in this case the problem of groundwater depletion (Pohl 2011). It can help inform dialogue with hydrogeologists to confront uncomfortable questions, challenges they face while being in the field, and practices of producing groundwater knowledge and lead to opportunities for co-producing groundwater knowledge. It necessitates a space for reflection as discussions with officials showed their cognisance of and contemplation of the challenges inherent in their work. This becomes crucial given that they work within significant institutional, budgetary, and political constraints and mandates from funding agencies like the World Bank.

## Conclusions

Situating socio-hydrogeology can help strengthen the participatory paradigm in groundwater governance. Participatory groundwater programmes, like ABhY discussed in this paper, have evolved, to integrate diverse interests and voices of groundwater user communities and other stakeholders (Ghose et al. 2018). However, while deemed participatory, these programmes reinforce epistemic hegemonies of how groundwater comes to be known- as seen in ABhY with the installation of piezometers and water level recorders to make groundwater knowable. As revealed in this paper, rural communities come to know groundwater (levels, conditions) through cultural and material relationships with groundwater and agriculture, while hydrogeologists, in their everyday practices, draw upon ‘beyond scientific’ knowledge of groundwater. As calls for improving and increasing citizen science in water and groundwater governance grow across the globe (UNESCO 2022), a *situated* socio-hydrogeological approach can enable re-imagining how we know and govern groundwater by moving beyond singular, disciplinary hegemonies towards a transdisciplinary praxis.

Groundwater depletion and associated challenges have led to calls for regional strategies (Aeschbach- Hertig and Gleeson 2012), that opens a scope for transforming how we- groundwater user communities, institutions, officials and policy makers, make groundwater knowable. Socio-hydrogeology, through its field-based, transdisciplinary framing of groundwater–society relationships, presents an opportunity to undertake this task through dialogue and partnerships between social and natural scientists. In the last decade, voices have been advocating for critical and reflexive consideration of scientific practices (Lane 2014; Tadaki et al. 2015), seeking a move away from a plug-in approach towards the social sciences. This paper, drawing on the insights emerging from critical water scholarship with an emphasis on *situating* socio-hydrogeology, attempts to shift the focus of invisibility from groundwater towards making visible the *social* in practices of groundwater knowledge production.

## Data Availability

The data that supports the findings of this study is available on reasonable request from the author (DJ). The data is not publicly available due to its sensitive nature, which may compromise the privacy of the research participants.
